# Ultra-Processed Food Consumption Is Associated with Abdominal Obesity: A Prospective Cohort Study in Older Adults

**DOI:** 10.3390/nu12082368

**Published:** 2020-08-07

**Authors:** Helena Sandoval-Insausti, Manuel Jiménez-Onsurbe, Carolina Donat-Vargas, Jimena Rey-García, José R. Banegas, Fernando Rodríguez-Artalejo, Pilar Guallar-Castillón

**Affiliations:** 1 Department of Preventive Medicine and Public Health, School of Medicine, Universidad Autónoma de Madrid-IdiPaz, CIBERESP (CIBER of Epidemiology and Public Health), 28029 Madrid, Spain; helenagabar@gmail.com (H.S.-I.); manuj.onsurbe@gmail.com (M.J.-O.); cdonatvargas@gmail.com (C.D.-V.); jimena.reygarcia@gmail.com (J.R.-G.); joseramon.banegas@uam.es (J.R.B.); fernando.artalejo@uam.es (F.R.-A.); 2 Department of Nutrition, Harvard T.H. Chan School of Public Health, Boston, MA 02115, USA; 3 IMDEA-Food Institute, CEI UAM + CSIC, 28049 Madrid, Spain; 4 Unit of Nutritional Epidemiology, Institute of Environmental Medicine, Karolinska Institutet, SE-171 77 Stockholm, Sweden; 5 Internal Medicine Department, Ramón y Cajal University Hospital, 28034 Madrid, Spain

**Keywords:** ultra-processed food, abdominal obesity, longitudinal study, seniors-ENRICA-1 cohort, NOVA classification

## Abstract

Introduction and objectives. Ultra-processed food (UPF) consumption has been associated with increased incidence of cardiovascular disease and its risk factors. The aim of this study was to assess, for the first time in the literature, the prospective association between UPF consumption and the incidence of abdominal obesity (AO) in older adults. Methods. The study sample consists of 652 participants in the Seniors Study on Nutrition and Cardiovascular Risk in Spain: Seniors-ENRICA-1 study, (mean age 67, 44% women). At baseline, standardized anthropometric measurements were collected (including abdominal circumference). After a median follow-up of six years, the abdominal circumference was measured again, and the incidence of abdominal obesity (AO) was calculated, defined as an abdominal perimeter ≥102 cm in men and ≥88 cm in women. At baseline, dietary information was collected using a computerized and validated dietary history. Information was obtained on the usual diet in the previous year. A total number of 880 foods were classified according to their degree of processing following the NOVA classification. Foods or drinks formulated mostly or entirely from substances derived from foods, with little or no presence of the unaltered original food were classified as UPF. For each participant, the percentage of energy from UPF was derived and sex-specific tertiles were calculated. Logistic regression models were built and adjusted for sociodemographic, lifestyle, morbidity, and drug treatment variables. Results. Among those participants without AO at baseline, 177 developed AO during follow-up. The average consumption of UPF was 17% of total energy (7% in the first tertile; 29% in the third tertile). The odds ratio (95% confidence interval) for incident AO risk when compared to the lowest tertile was: 1.55 (0.99–2.44) for the second tertile of UPF consumption and 1.62 (1.04–2.54) for the third tertile; *p* for linear trend: 0.037. Results remained statistically significant after adjusting for potential dietary confounding factors such as fiber consumption, the intake of very long chain omega-3 fatty acids and adherence to the Mediterranean diet. Conclusions. A higher UPF consumption is positively associated with incident AO in older adults in Spain. These findings extend the current evidence of the detrimental effect of UPF consumption on cardiometabolic health.

## 1. Introduction

In recent decades, the food industry has elaborated products characterized by their great taste, ready availability, long shelf life, affordability and that are microbiologically safe. Some of these foods are ultra-processed foods (UPF), and the NOVA classification has been widely used for their identification. The NOVA classification categorizes foods according to the extent and purpose of their industrial processing. According to this classification, UPF is the group that includes foods or drinks formulated mostly or entirely from substances derived from foods, with little or no presence of the unaltered original food. The NOVA classification also provides the population with an instrument that easily distinguishes whether a food is UPF or not. Therefore, the NOVA classification may be useful for both, research and food choices for the general population [1,2,3].

UPF is characterized by being rich in simple sugars, fats and salt, as well as being poor in fiber and micronutrients [4]. They also contain a large number of additives such as flavorings (e.g., phosphates), sweeteners, emulsifiers, colorings, wetting agents, hardeners, etc. In addition, these products are attractively packaged and aggressively marketed to supply a growing market [2]. As a result, UPF consumption has grown in all countries, having exceeded 50% of energy consumption in some Northern European countries [5], and 25% in some developing countries [6]. In Spain, UPF consumption is a major concern due to the fact that, at the same time the consumption of UPF is increasing, there is a decrease in the adherence to the Mediterranean diet, currently considered the healthiest diet in the world [7].

Several ecological studies have shown that parallel to the increase in UPF consumption there has also been an increase in the prevalence of general obesity [6,8], and so, UPF is now considered an important contributor to the current obesogenic environment. UPF consumption has also been associated with being overweight or having general obesity in several cross-sectional [9,10] and longitudinal studies [11,12].

In older adults, abdominal obesity (AO) is a risk factor for cardio–metabolic diseases and acts independently from general obesity [13], but very few studies have assessed the association between UPF consumption and AO in this population. To our knowledge there is only one cross-sectional study conducted in Brazil with 8977 middle-aged participants that found a positive association between UPF consumption and abdominal circumference [11], and a cross-sectional study that found an association between UPF consumption and metabolic syndrome as well as excess weight in US adults [14].

Therefore, the aim of this study was to assess, for the first time in the literature, the prospective association between UPF consumption and incident AO in non-institutionalized older adults.

## 2. Participants and Methods

### 2.1. Study Population

Participants came from the Seniors-ENRICA-1 cohort, which was established during 2008–2010 with participants aged 60 and over in Spain. Data were collected by trained and qualified personnel in 3 consecutive stages. At baseline, a telephone interview was conducted to collect sociodemographic variables, lifestyles, cardiometabolic risk factors, warning signs and symptoms of cardiovascular disease, as well as health status. Next, two home visits were conducted to obtain blood and urine samples, measure anthropometric parameters and blood pressure and obtain dietary information. In 2015, another wave of data collection was performed and included a telephone interview and one home visit.

**Figure 1:**
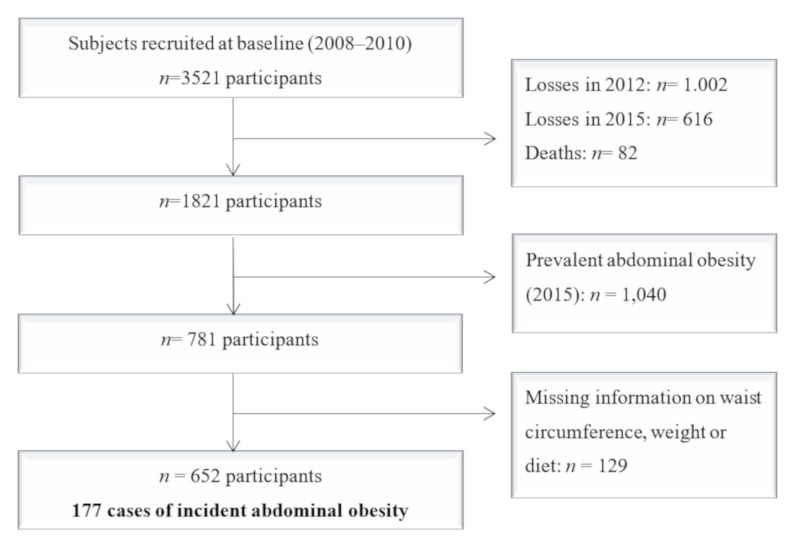
Flow chart of participants.

Of the 3521 participants in the baseline sample, 1618 were excluded due to loss to follow-up in the years 2012 and 2015 (1002 and 616, respectively) and 82 due to death. Of the remaining 1821, 1040 were excluded because they had prevalent AO at baseline, defined according to the WHO criteria (abdominal circumference ≥102 cm in men and ≥88 cm in women). We also excluded 129 participants with no data for waist circumference or extreme data in energy consumption (total energy intake out of range: 800–5000 kcal/day in men or 500–4000 kcal/day in women). Finally, the study sample consisted of 652 participants, of which 177 developed incident AO (Figure 1).

The study was approved by the Clinical Research Ethics Committee of the La Paz University Hospital. All participants gave their informed consent in writing.

### 2.2. Study Variables

#### 2.2.1. UPF Consumption and AO Assessment

Habitual food consumption was ascertained with a face-to-face dietary history (DH-ENRICA), recording all food consumed in a typical week in the preceding year. It was validated against seven 24-hour recalls in the preceding year, obtaining high correlation coefficients [15]. DH-ENRICA was developed base on the dietary history used in the EPIC cohort and included more than 860 foods, 30 different cooking methods and 120 photograph sets to help the participants to choose the serving sizes. Trained and certified interviewers recorded the data. Food consumption for each participant was classified into 4 groups by applying the NOVA classification [2,16]. The first group included unprocessed or minimally processed foods. These are natural foods without added substances and directly extracted from nature, which may or may not undergo minimal processing in order to preserve them better, prolong their viability and availability in stores; they are edible parts of plants, animals, fungi, algae or water (e.g., fruit, vegetables, cereal, seeds, fresh meat and fish, etc.). The second group included processed culinary ingredients. These are products that come directly from nature or are derived from group 1 and are used to preserve and extend the shelf life or to cook other foods from group 1 (e.g., oil, vinegar, honey, salt, sugar and butter). The third group is processed food that results from combining and processing fresh or minimally processed foods with foods from group 2 in order to increase their shelf life and/or modify their sensory qualities (e.g., canned vegetables, canned meats and fish, fruit in syrup, etc.). Finally, the fourth group consists of UPF. This group includes foods or drinks formulated mostly or entirely from substances derived from foods, with little or no presence of the unaltered original food (e.g., soft drinks, snacks, cookies, candies, ice cream, pizzas, instant soups, ultra-processed meat, etc.). See the Supplementary Materials for more details.

Waist circumference was measured by qualified personnel at baseline (years 2008–2010) and at the end of follow-up (year 2015), during physical examinations at home visits. Incident AO was calculated according to WHO criteria as waist circumference ≥102 cm in men and ≥88 cm in women in 2015, among those without AO at baseline.

#### 2.2.2. Potential Confounding Variables

At baseline, we collected sociodemographic and lifestyle variables such as age, sex, educational level (primary education or less, secondary education and university), marital status (married, not married), smoking, ex-drinker status, physical activity in the household, physical activity during leisure time, number of medications consumed daily (as a proxy for morbidity), as well as the prevalence of chronic diseases diagnosed by a physician (chronic obstructive pulmonary disease/asthma, coronary heart disease, stroke, heart failure, osteoarthritis, cancer or depression).

As proxies for a good diet quality, analyses were also controlled for fiber and intake of very long-chain ω-3 fatty acids, as well as for adherence to the Mediterranean diet. This latter was assessed with a modified Mediterranean index based on the one used in the PREDIMED study [17], but we excluded the items with UPF in their definition, so that, for the purpose of our work, the modified Mediterranean index only included the following 8 items: Do you use olive oil as the principal source of fat for cooking? How much olive oil do you consume per day (in spoons)? How many servings of vegetables do you consume per day? How many pieces of fruit do you consume per day? How many servings of pulses do you consume per week? How many servings of fish or shellfish/seafood do you consume per week? How many servings of nuts do you consume per week? Finally, do you prefer to eat chicken, turkey or rabbit meat instead of beef, pork, hamburgers or sausages?”

#### 2.2.3. Statistical Analysis

The percentage of energy from UPF over the daily total energy intake was calculated for each participant, and the sample was divided into sex-specific tertiles. Binary logistic regression models were used to assess the association between UPF consumption and incident AO. The first (lowest) tertile was considered as reference and odds ratios (OR) and their 95% confidence intervals (CI) were obtained. The *p* for linear trend was calculated using the tertiles of the percentage of UPF consumption modeled as a continuous variable.

We built four logistic regression models with consecutive adjustment levels: model 1 was adjusted for sex and age; model 2 was further adjusted for sociodemographic variables (educational level and marital status) and lifestyles (smoking, ex-drinking status, physical activity in the household and leisure time physical activity); model 3 was further adjusted for clinical factors (number of medications per day and number of chronic diseases); Finally model 4 was additionally adjusted for dietary variables (fiber and very long chain omega-3 fatty acid consumption as well as adherence to the Mediterranean diet). Furthermore, we carried out a sensitivity analysis on model 4 by adjusting for total energy consumption according to the energy partitioning method [18].

The analyses were performed with STATA software version 14 for Windows (StataCorp LP, College Station, TX, USA), setting the statistical significance at *p* < 0.05.

## 3. Results

Of the 652 participants selected for the analyses (55.7% men; mean age 67.1 ± 5.8 years), 177 developed AO after 6 years of follow-up. The average percentage of energy consumption from UPF was 17.3% in the whole sample (7.3% in the first tertile; 16.0% in the second tertile and 28.7% in the third tertile). Participants in the highest tertile of UPF intake also consumed more total energy, had lower intake of very long chain omega-3 fatty acids and less adherence to the Mediterranean diet when compared with participants in the first (lowest) tertile (Table 1).

In the multivariable analyses, participants with a higher UPF consumption were more likely to develop AO. The OR (95% CI) for the risk of AO was 1.55 (0.99–2.44) for the second tertile and 1.62 (1.04–2.54) for the third tertile when compared to the first tertile of UPF consumption (*p* for linear trend: 0.037) (Table 2, model 3).

Results remained statistically significant after adjusting for nutritional variables such as fiber intake, intake of very long chain omega-3 fatty acids and adherence to the Mediterranean diet (Table 2, model 4). A sensitivity analysis was carried out by adjusting for total energy intake and, again, similar results were obtained; the ORs for AO were 1.84 (1.05–3.23) for the second tertile and 2.55 (1.04–6.27) for the third tertile when compared to the lowest UPF consumption tertile (*p* for linear trend: 0.032).

**Figure 2:**
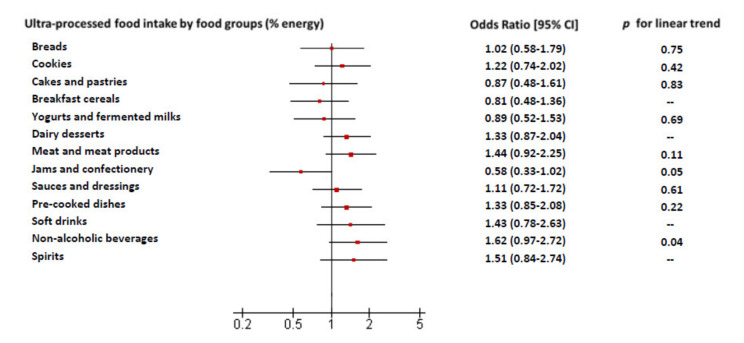
Odds ratio of abdominal obesity risk and 95% confidence intervals (95% CIs) for tertile (highest intake) of groups of ultra-processed food consumption as a percentage of total energy (% of energy) versus Tertile 1 (lowest intake), in the Seniors-ENRICA 1 cohort study. *n* = 652. When the intake of ultra-processed food from a specific food group occurred in less 25% of the participants, the odds ratio (95% CI) was calculated between subjects who consumed the food compared to those who did not (as in breakfast cereals, dairy desserts, soft drinks and spirits). *p* for linear trend was calculating using tertiles as a continuous variable. Model was adjusted for sex, age, level of education, marital status, tobacco consumption, ex-drinker status, physical activity at household, physical activity at leisure time, number of medications used, number of chronic diseases (respiratory disease, coronary disease, stroke, osteoarthritis/arthritis and depression), fiber intake, long-chain ω-3 fatty acids intake and Mediterranean diet adherence. Other non-alcoholic beverages group includes instant coffee drinks and cocoa drinks, packaged juices and other non-alcoholic drinks, excluding soft drinks.

The food groups that contributed the most to this association were non-alcoholic beverages (including instant coffee drinks, cocoa drinks and packaged fruit juices), spirits, meat products and soft drinks. However, none of these UPF subgroups achieved statistical significance when comparing individually the extreme tertiles. Non-alcoholic beverages showed a significant linear trend when including tertiles as a continuous variable in the models (Figure 2).

## 4. Discussion

Older adults from Spain had relatively low UPF consumption (17.3% of total energy), although there was a wide variation indicating heterogeneous consumption habits in this population. After six years of follow-up, higher UPF consumption was associated with an increased risk of AO. This increased risk of AO was observed with relatively low consumption of UPF (average consumption 28.7% in the highest tertile). This association was maintained after adjusting for some healthy dietary variables such as dietary fiber consumption, intake of very long chain omega-3 fatty acids from fish, adherence to the Mediterranean diet and energy consumption, indicating that UPF consumption could also act thorough other additional mechanisms.

The association between UPF consumption and AO was previously assessed in a cross-sectional study conducted on a sample of middle-aged participants from Brazil. They had a UPF consumption average of 22.7% (slightly higher than in this study), and a higher UPF consumption was associated with a higher waist circumference [11]. We found no other studies that looked at UPF consumption as a whole, but there is evidence that some UPF groups are individually associated with AO. For example, a longitudinal analysis of the PREDIMED study found that in patients with high cardiovascular risk, increased consumption of some UPF (e.g., ultra-processed meat) was associated with increased waist circumference. However, in this study, most foods were not classified according to their degree of processing and UPF consumption was not assessed as a whole [19].

There are several potential mechanisms for the association between UPF and AO. UPF have a high energy density that delays the signals of satiety [2], and larger portions of these foods are usually consumed [20]. Another mechanisms could be their low nutritional quality, with a high content of saturated fatty acids, trans-fatty acids, refined sugars and low content of fiber, vitamins and antioxidants [21]. In addition, new substances are generated in food processing, for example during packaging, moisture removal, heat treatments, cooling and freezing, acidity control and irradiation. A well-known example is phthalates, which are present in plastic packages and could act as endocrine disruptors after food contamination [22]. Finally, additives are of particular concern [23]. Some of them, such as sulfites or phosphates have shown adverse effects on cardiovascular health [24].

The social and health relevance of AO development in the older adult population is enormous. First, because 61.6% of the older adults in Spain already have AO, with this prevalence being even higher among women (69.7%) [25]. Second, because this population already has many cardiovascular risk factors including tobacco consumption, low physical activity, high total cholesterol levels, high blood pressure and impaired glycemic metabolism, with very little reaching of ideal cardiovascular metrics [26]. Third, because AO is associated with increased risk of disability, mainly related to mobility and agility, and this risk is independent of BMI [27]. Fourth, because increased fat mass in this population may be associated with sarcopenic obesity [28]. Fifth, because UPF consumption has been associated with frailty syndrome in this population [3]. Moreover, finally, because once AO is established, it is very difficult to reverse it at older ages.

This study expands the evidence of the harmful effects of UPF consumption on cardiometabolic health. Previously, UPF consumption has been associated with increased overweight and general obesity [10], hypertension [29], dyslipidemia [30], diabetes [31], incident cardiovascular diseases [32], depression [33], and all-cause mortality [34]. Gathering evidence on the deleterious effect of UPF consumption is important because it is a universal and ubiquitous exposure, affecting all ages. In addition, we unfortunately do not know the influence of this prolonged exposure over time, but it is possible to venture that some UPF substances that act as endocrine disruptors may have a role in increasing metabolic diseases in the future. Further research should move towards understanding the specific components and in particular UPF food subgroups that are most associated with an increased risk of disease.

Government agencies have begun to take some Public Health measures regarding UPF consumption. For example, the implementation of the Nutri-score labeling on the front of packaging is a step in the right direction. This labeling has been shown to increase the nutritional quality of the shopping basket, helping consumers make healthier choices [35]. However, some of these proposals are based only on nutritional composition and do not take into account other chemicals or additives found in UPF. Another important step will be the validation of mobile applications that allow you to know the degree of processing of the products and their content in deleterious health additives. Many of these initiatives require the use of a system of product codes that could be standardized in the future. The industry must also make economic and production changes to offer healthier food to consumers. These changes are often brought about by the implementation of regulations at a national or an international level [36] or by significant social pressure. There is a growing social awareness that calls for the possibility to make healthy choices for consumers and this social awareness is expected to increase in the future. In addition, there are positive initiatives such as “power” (“poder” in Spanish) that proposes changes in advertising, supply, demand, labeling and food reformulation [37].

This research has several limitations. First, although the NOVA classification is the most widely used, it is not without its critics, because of possible inaccuracies in UPF classification [38]. However, we think that these inaccuracies may lead to an erroneous non-differential misclassification in the exposure that biases towards the null. Furthermore, our data collection instrument allows us to distinguish foods according to the processing they have undergone, and therefore we think this error is potentially small. On the other hand, this classification is easy to understand for an older person who must make the purchase, which in turn facilitates the communication of the results to society. Additionally, it allows for comparison with other studies that have used the same classification. Second, the number of people who have developed incident AO was low, in part because of the enormous number of participants who were already suffering from AO at the beginning of the study. Thus, although the statistical power was sufficient to find statistically significant relationships, it has not allowed us to disaggregate by sex, and the power for a subgroup study is limited. Third, repeated dietary measurements were not taken into account, and it is possible that an increase in UPF consumption happened during follow-up. It is assumed that diet worsens over time due to marketing and the increased availability of UPF, and subsequently this lack of precision in the exposure assessment may bias to the null. Finally, we cannot rule out some degree of residual confounding.

Among the strengths of our study are the longitudinal design as well as the initial use of a representative sample of older adults from Spain. In addition, the use of standardized protocols and trained personnel for the collection of anthropometric variables reduces classification bias. Finally, numerous confounding variables were used in the adjustment yielding robust results in different models.

In conclusion, UPF consumption is associated with an increase in the incidence of AO in the non-institutionalized Spanish population of older adults. This finding extends the accumulated evidence of the detrimental effects of UPF on cardiometabolic health, and they should stimulate the food industry to decrease the obesogenic nature of the products they develop.

## Figures and Tables

**Table 1:** Baseline sociodemographic and lifestyle characteristics of participants according to sex-specific tertiles of ultra-processed food consumption (% of energy) in the Seniors Study on Nutrition and Cardiovascular Risk in Spain: Seniors-ENRICA 1 Study (2008–2010).

Ultra-Processed Food Consumption (% of Total Energy)					
Characteristics	Total (*n* = 652)	Tertile 1 (Lowest) (*n* = 217)	Tertile 2 (*n* = 217)	Tertile 3 (Highest) (*n* = 218)	*p* for Linear Trend
Total energy (kcal/day), mean ± SD	2052 ± 561	1954 ± 530	2062 ± 566	2142 ± 573	<0.001
Consumption of ultra-processed foods (% of energy), mean ± SD	17.3 ± 10.17	7.25 ± 3.24	16.02 ± 2.87	28.69 ± 7.66	<0.001
Women (%)	44.3%	44.7%	44.2%	44.0%	0.89
Age, mean ± SD	67.08 ± 5.8	67.4 ± 6.06	67.04 ± 5.37	66.8 ± 5.99	0.28
Educational level (%)					0.86 *
Primary	40.5%	41.0%	42.9%	37.6%	
Secondary	29.9%	29.5%	28.1%	32.1%	
University	29.6%	29.5%	29.0%	30.3%	
Married (%)	75.5%	75.6%	74.7%	76.2%	0.89
Tobacco consumption (%)					0.08 *
Smokers	14.6%	11.1%	15.7%	17%	
Ex-smoker	29.9%	31.8%	26.7%	31.2%	
Non-smoker	55.5%	57.1%	57.6%	51.8%	
Ex-drinker, (%)	7.7%	10.6%	3.2%	9.1%	
Physical Activity (METs-h/week), mean ± SD					
In the household	35.18 ± 28.81	37.17 ± 30.96	33.10 ± 27.03	35.31 ± 28.31	0.50
At leisure time	24.61 ± 16.10	25.24 ± 16.83	24.50 ± 15.6	24.10 ± 15.90	0.46
Number of medications per day, mean ± SD	1.46 ± 1.63	1.48 ± 1.63	1.55 ± 1.73	1.36 ± 1.54	0.46
Number of chronic diseases (%), mean ± SD	0.52 ± 0.64	0.55 ± 0.66	0.49 ± 0.62	0.52 ± 0.64	0.68
Fiber (g/day), mean ± SD	25.31 ± 8.48	26.10 ± 9.40	25.13 ± 8.32	24.73 ± 7.62	0.10
Very long chain omega-3 fatty acids (g/day), mean ± SD	1.02 ± 0.88	1.17 ± 1.11	1.01 ± 0.77	0.88 ± 0.70	<0.001
Adherence to the Mediterranean diet **, mean ± SD	3.02 ± 1.32	3.44 ± 1.40	3.03 ± 1.21	2.60 ± 1.20	<0.001

SD: standard deviation; * Pearson’s chi-squared was calculated. ** Adherence to the Mediterranean diet was calculated with a modified Mediterranean index including 8 items that did not have ultra-processed food in their definition. The cutoff points for tertiles of the percentage of energy from ultra-processed food were: Tertile 1 (0.14–12.4), Tertile 2 (12.5–22.4), Tertile 3 (22.5–62.2) in men; Tertile 1 (0–10.5), Tertile 2 (10.6–19.30), Tertile 3 (19.31–57.5) in women.

**Table 2:** Risk of abdominal obesity according to tertiles of ultra-processed food consumption in the Seniors-ENRICA 1 study.

Ultra-Processed Food Consumption (% of Energy)				
	Tertile 1 (Lowest)	Tertile 2	Tertile 3 (Highest)	*p* for Linear Trend
*n*	217	217	218	
Abdominal obesity cases	46	65	66	
Model 1, OR (95% CI)	1 (Ref.)	1.62 (1.04–2.51)	1.64 (1.06–2.56)	0.029
Model 2, OR (95% CI)	1 (Ref.)	1.58 (1.00–2.47)	1.61 (1.03–2.51)	0.041
Model 3, OR (95% CI)	1 (Ref.)	1.55 (0.99–2.44)	1.62 (1.04–2.54)	0.037
Model 4, OR (95% CI)	1 (Ref.)	1.54 (0.98–2.44)	1.61 (1.01–2.56)	0.048

OR: odds ratio; CI: confidence interval; Model 1: adjusted for age and sex; Model 2: adjusted as in model 1 plus educational level, marital status, smoking, ex-drinker status, physical activity in the household and at leisure time; Model 3: adjusted as in model 2 plus the number of medications consumed per day and the number of chronic diseases diagnosed by a doctor (chronic obstructive pulmonary disease/asthma, coronary heart disease, stroke, heart failure, osteoarthritis or depression); Model 4: adjusted as in model 3 plus daily intake of fiber, intake of very long chain omega-3 fatty acid and the 8-point index of adherence to the Mediterranean diet.

## Supplementary Materials

The following are available online at https://www.mdpi.com/2072-6643/12/8/2368/s1, Supplementary Material: Total food items considered in the ENRICA study (2008-10) according to the extent of processing (NOVA classification).
