# Supplementary material for: Ultra-Processed Food Consumption Is Associated with Abdominal Obesity: A Prospective Cohort Study in Older Adults
Source: Nutrients. 2020 Aug 7;12(8):2368. doi: 10.3390/nu12082368 (PMC7468731; doi:10.3390/nu12082368)
Supplement: Supplementary file 1 [file nutrients-12-02368-s001.pdf]

Supplemental Appendix. Total food items considered in the ENRICA study (2008-10) according to the extent of processing (NOVA classification).

1) Unprocessed or minimally processed foods

Cereals:
- *Grains and flours*: Boiled white rice, boiled whole grain rice, corn flour, gofio (flour from roasted grains), rye flour, wheat flour, whole grain wheat flour, quinoa, wheat semolina, boiled couscous, corn flour.
- *Pasta*: boiled pasta (macaroni, spaghetti).
- *Breakfast cereals*: oat bran, wheat bran.

Milk and dairy products:
- *Milk*: whole milk, whole goats milk, sheep's milk, skimmed milk (0-1%), powdered skimmed milk, whole dry milk, low fat powdered milk (2%), whole milk (4%), whole fresh milk, semi skimmed milk (2%).
- *Yogurts and fermented milks*: natural skimmed fermented milk, natural fermented whole milk, natural skimmed yogurt, natural whole yogurt, kefir/ kephir milk.

Meat and meat products
- *Pork*: pork (ground meat with lard/bacon fat), lean pork, pork chops with fat, lean pork chops, pork ribs, pork fillets, pork sirloin, pork trotters.
- *Veal*: veal, lean veal, veal chops, veal ribs, veal rack, lean veal rump steaks (entrecote), veal shoulder blade steaks, veal round steaks, veal sirloin steaks.
- *Lamb*: lamb, lean lamb, lamb chops, lambs head, lean shoulder blade of lamb, leg of lamb.
- *Beef:* beef, beef rib-eye (entrecote), beef skirt steaks, boneless beef eye, beef sirloin steaks.
- *Poultry*: chicken, whole snipe, hens, skinless hens, skinless goose, whole duck, skinless duck, turkey (legs/drumsticks), skinless turkey legs/drumsticks, turkey breasts, skinless turkey, skinless pigeon, whole chicken, chicken drumsticks, skinless chicken drumsticks, whole chicken breasts, skinless chicken breasts, skinless chicken, partridge.
- *Offal*: offal, tripe, cows heart, lamb's liver, chicken liver, ox liver, veal's tongue, calf's sweetbreads, pig's kidneys, lamb's kidneys, calf's kidneys, pig's brain, lamb's sweetbreads.
- *Other meats and meat products*: horse meat, kid (goat), rabbit, wild pig (boar).

Eggs and egg products
Raw hen egg whites, hens egg, lightly pouched hen's eggs, fried hen's eggs, hard-boiled eggs, pouched hen's eggs, raw hen egg yolks, snipe's eggs.

Fish and fish products
- *White fish*: fillets of white fish, white fish, haddock, blue whiting, cod, red bream, scorpion fish, dogfish/shark marinated in vinegar and oil, black sea bream, ribbon fish, gilt-head bream, bay whiff, halibut, sole, seabass, whiting, hake, frozen hake, grouper fish/sea perch, grey mullet, Breca fish/Pandora fish, small whiting/young hake, small frozen whiting, catfish, flounder, monkfish, skate/ray, turbot, red mullet, white seabream, shark.
- *Blue fish*: blue fish, eel, tuna, albacore/longfin tuna, bonito (tuna), anchovy, mackerel, whitebait, conger eel, emperor fish, pompano fish, blue whiting, red bream/red porgy, small fried whitebait, swordfish, salmon, sardines, baby eels.

- *Freshwater fish*: pike, perch, trout.
- *Other fish and fish products*: caviar from sturgeon/roe, lumpfish roe, dried salted tuna.

Seafood
- *Crustaceans*: lobsters, crabs, freshwater crayfish, spider crabs, langoustine/Dublin bay prawns, prawns/shrimp, frozen prawns/shrimp, baby shrimp, rock lobsters, jumbo shrimp, small crabs, barnacles/goose barnacles.
- *Molluscs*: clams, cockles, winkles, squid, sea snails, baby squid, baby clams, mussels, razor shells/razor clams, oysters, octopus, cuttlefish, scallops.

Vegetables
- *Steam and leaf vegetables:* chard/Swiss chard, celery, watercress, cabbage, borage (starflower), cardoon, chives, white cabbage, Brussels sprouts, red cabbage, green cabbage, endives, curly endives, raw asparagus, boiled green asparagus, cooked spinach, raw spinach, green turnips, fennel, lettuce.
- *Root vegetables*: parsnips, radishes, beetroot, soya sprouts/soya shoots, carrots.
- *Bulbs vegetables*: garlic, green garlic/garlic shoots, onions, leaks,
- *Inflorescence vegetables and fruits*: artichokes, aubergines, broccoli, courgettes, pumpkin, cauliflower, frozen cauliflower, frozen green beans, boiled green beans, corn, cucumbers, peppers, red peppers, green peppers, tomatoes.
- *Mushrooms*: mushrooms, milk cap mushrooms/red pine mushrooms, brewer's mushrooms/wild fungus/edible fungus.

Legumes and legume products
- *Fresh legumes*: frozen boiled peas, boiled peas, broad beans.
- *Pulses*: boiled white beans, boiled black beans/frijoles, boiled chickpeas, beans, boiled lentils, boiled soya, boiled beans, boiled canned beans.
- *Legume's derivate products*: soya flour, soya milk.

Tubers and tuber products
Potatoes, roasted potatoes, boiled potatoes, sweet potatoes, tapioca, yams.

Fruits and fruit products
- *Fresh fruit*: apricots, cranberry, kaki, cherries, apple custard, whole prunes, raspberries, strawberries, pomegranate, gooseberries, prickly pears, figs, kiwis, litchi, limes, lemons, fresh lemon juice, mandarines/tangerines, mango, whole roast apples, whole apples, peeled golden delicious apples, peeled apples, peeled starking apples, fruit, peaches, melons, quince, blackberries, oranges, fresh orange juice, nectarines, peeled medlar fruit, papayas, Paraguayan, whole pears, peeled pears, pineapples, bananas, grapefruit, fresh grapefruit juice, watermelon, black grapes, green grapes.
- *Dried fruits*: dried apricots, dried plums, dried dates, dried figs, dried peaches, raisins
- *Oleaginous fruits*: avocado.

Nuts
Nuts, raw almonds, toasted almonds, hazelnuts, chestnuts, toasted chestnuts, raw tigernuts, fresh coconuts, dried coconuts, nuts, peanuts, unsalted sunflower seeds, sesame seeds, cashew nuts, macadamia nuts.

Non-alcoholic beverages
- *Water*: bottled sparkling water, water/tab water, mineral water, still water,

- *Coffee and brews*: decaffeinated coffee made with an Italian coffee maker, decaffeinated expresso, filtered decaffeinated coffee, expresso made with an Italian coffee maker, expresso, filtered coffee, tea/herbal tea, tea with milk, tea, wheat bran.

Miscellanea
Fresh yeast.

2) Processed culinary ingredients

Oils and fats
- *Oils***:** oil, seed oil, canola oil, sunflower oil, sunflower and olive oil 50%, corn oil, olive oil, olive oil (extra virgin), soya oil.
- *Fats*: lard, butter.

Sugars and sugary products
- *Sugars*: White sugar, brown sugar, fructose, honey.

Condiments:
Cinnamon, chili peppers/chili peppers, fresh parsley, paprika, ground pepper, table salt, vinegar.

3) Processed foods

Cereals
- *Breads:* White bread, biscotte/rusk bread/breadsticks, french stick, french stick without salt, white sliced bread, french stick/baguette, loaf of bread/ciabatta, mixed wheat and rye bread, whole grain bread

Milk and dairy products
- *Fresh cheeses*: coat's cheese, fat free fromage frais, mixed goat and cows's cheese, fresh cheese in tubs, mozzarella cheese (buffalo milk), fresh coat's cheese, cottage cheese,
- *Cured cheeses*: semi-cured cheese, soft cheese, blue cheese, babybel cheese/edam cheese, brie cheese, camembert 20-30% fdm (fat content in dry matter), camembert 40-50% fdm (fat content in dry matter), camembert 60% fdm (fat content in dry matter), aged goat's cheese, cheddar cheese, soft cow's cheese, edam cheese, goat's cheese with mould, cured goat's cheese, feta cheese (ewe and goat's milk), semi-cured goat's cheese, blue cheese, sheep and goat's cheese, sheep, cow and goat's cheese, sheep and cow's cheese, edam cheese, emmenthal cheese, gouda cheese, gruyere cheese, unpasteurized goat's cheese, soft to hard white cow's milk cheese, goat's cheese (milky and nutty flavour), manchego cheese/sheep's cheese, cured manchego cheese/cured sheep's cheese, sheep's cheese in oil, semi-cured sheep's cheese, muenster cheese, cream cheese, parmesan cheese, goat's cheese, sheep's cheese, roquefort cheese, cow's cheese, tetilla cheese (cow's milk), torta del casar cheese (sheep's milk), zamorano cheese (hard sheep's cheese), daised semi-cured goat's cheese, daised cured goat's cheese, mould daised ripened semi-cured goat's cheese, diced fresh cheese, diced semi-cured cheese, diced cured cheese.

Meat and meat products
- *Cold meats*: corned beef/dried beef, ham cured in salt with fat/tocino, ham cured in salt without fat/tocino, dried pork sirloin.
- *Other* m*eat products*: bacon, smoked bacon, homemade hamburgers, homemade grounded/minced meat, stewed hare with sauce, fried blood with onions.

Eggs and egg products
Scramble eggs, French omelette.

Fish and fish products
Smoke fish, salted fish, salted cod, small fried whiting, anchovies in oil, kippers, canned albacore in oil, canned natural tuna, canned tuna in oil, canned tuna marinated in vinegar, canned natural bonito (tuna), canned bonito in oil, canned bonito cooked and marinated in vinegar, anchovies in vinegar, canned mackerel in oil, smoked salmon, canned sardines in oil, canned marinade sardines, smoked trout.

Seafood
Canned natural crabs, natural canned cockles, canned marinated mussels.

Vegetables
Canned celery, canned white asparagus, palm hearts, canned soya sprouts, canned carrots, canned beetroot, canned green beans, canned boiled corn, canned tomatoes, canned artichokes, red bell peppers, canned mushrooms, mixed canned vegetables/diced vegetables.

Legumes and legume products
Canned peas, canned white beans, canned chickpeas, canned lentils, tofu.

Tubers and tuber products
Homemade fried potatoes.

Fruits and fruit products
- *Fruits preserves*: Fruit in syrup, guava in syrup, fruit cocktail in syrup, peaches in syrup, pears in syrup, pineapple in syrup, pineapple in natural juice.
- *Oleaginous fruits*: olives, black olives, green olives.

Nuts
Fried salted almonds, fried salted peanuts, nuts and raisins, salted toasted pistachio nuts, salted toasted peanuts, roux.

Sauces and dressings
Natural fried tomatoes sauce.

Homemade cooked food
Homemade lasagna, homemade lasagna with bechamel, sautéed peppers, onions and tomatoes, fish, onion soup, tapioca soup/tapioca dips.

Snacks
Capers, lupines, large capers, canned baby or pearl onions in vinegar, pork crackling snacks, mixed pickles, toasted chickpeas, canned pickles in vinegar/ pickled gherkins.

Non-alcoholic beverages
Almond milk, natural tomato juice.

Alcoholic beverages

Beer, stout beer, lager, cider, sweet cider, dry cider, wine, sparkling wine/champagne, sparkling very dry white wine, white wine, sherry, rosé wine, red wine, brewer's yeast/beer yeast.

4) Ultra-processed food and drink products

Cereals:
- *Pasta*: boiled pasta with eggs, meet ravioli, cheese ravioli.
- *Breads:* crispbread, whole grain crispbread, white sliced bread, hamburger buns, hot dog buns, Vienna style loaf of bread, brown sliced bread, bread crumbs, pizza bases, croutons.
- *Cookies*: biscuits, chocolate chip cookies, chocolate filled biscuits, tea biscuits, whole grain crackers, tea biscuits.
- *Cakes and pastries*: tea cakes, pastries, whole grain pastries, cake, lady fingers/sponge fingers/sponge cake, tea buns/soft bread buns, chocolate filled buns, custard filled buns, cream buns, Swiss buns, choux buns, chocolate Danish pastry, churros, dried fruit sponge cake, croissant, chocolate filled croissants, cheese filled croissants, doughnuts, Mallorcan Danish pastry, fairy cakes/cupcakes, short crust pastry, puff pastry, lard, meringue, puff pastry filled with crème and custard, Danish pastry, puff pastry, Danish pastry filled with jam, chocolate cake, sponge cake, cream cake, pastry filled with custard, plum cake, homemade doughnuts, cake filled with cream, fruit cake, whisky cake, apple pie, cheese cake, egg pudding, dried fruit sponge cake, sponge cake, chocolate sponge cake, tea buns, butter sponge cake, Madeira cake, choux pastry with custard, fruit Danish pastry, raisin pudding, apple pie with custard.
- *Breakfast cereals*: cereals, puffed rice/rice krispies, bran flakes/all-bran, chocolate cereal flakes, crunchy/honey cereal flakes, corn flakes, muesli, puffed wheat.

Milk and dairy products:
- *Milkshakes*: chocolate milk, milkshakes, sweet condensed milk, evaporated milk/carnation milk, cooking cream.
- *Yogurts and fermented milks*: skimmed fermented milk with fruits, whole fermented milk with fruits, skimmed flavoured yogurt, skimmed yogurt with fruits, natural skimmed yogurt with sweetener, whole flavoured yogurt, whole yogurt with fruits, natural whole yogurt with sweetener, whole drinking/liquid yogurt, whole drinking/liquid yogurt with fruit, natural drinking/liquid yogurt with sweetener, natural Greek yogurt.
- *Dairy desserts*: custard, pastry cream, curd, egg flan with crème caramel, vanilla flan, chocolate ice cream, creamed ice cream, vanilla ice cream, ice cream, flavoured commercial mousse, custard, cream brulée, chocolate mousse, chocolate custard, natural petit Suisse with sweetener.
- *Ultra-processed cheeses* flavoured petit Suisse, babybel cheese/edam cheese, raclette cheese (semi-hard cow's milk), sliced cheese, processed cheese in portions, processed light cheese, cheese spread/cheese dip, extra creamy melting cheese, creamy melting cheese, cheese spread, soft cheese type Philadelphia/cheese spreads, grated parmesan cheese, light cheese.
- *Creams:* whipping cream, whipping cream with sugar.

Meat and meat products
- *Meats:* ground/minced meat, pork (ground/minced meat), ground/minced veal.
- *Cold meats:* chistorra, chorizo/dried sausages, fresh chorizo/fresh sausages, chorizo, salami, dried sausages, pork sausages, chorizo, sausage, chorizo spread, cold meats,

bratwurst, Catalan sausages, wild pig (boar) jellied sausage, cold meat galantine, foie-grass, boiled ham, shoulder of Ham, blood pudding/black pudding, blood pudding/black pudding, cold sausage type salami, smoked pancetta, pigs liver-pate, turkey slices, salami, Frankfurt sausages, Catalan sausages, blood pudding with rice, champagne pate/country pate.
- *Other meat products*: pork meatballs, meatballs, seasoned ground/minced meat for stuffing, chicken hamburgers, beef hamburgers, restaurant/industrial hamburgers, chicken sausages, fresh sausages, canned meatballs.

Fish and fish products
Surimi/crab sticks, surimi.

Oils and fats
- *Fats:* Margarine (75% from vegetables), light margarine, mixed margarine (50% from animals, 50% from vegetables), vegetable margarine.

Legumes and legume products
Soya milkshake.

Tubers and tuber products
Potato puree made with milk, potato puree made with water.

Fruits and fruit products
Apple compote with sugar/stewed apples with sugar.

Nuts
Peanut butter.

Sugars and sugary products
- *Jams and confectionery:* jam, low calorie jam, sweets/candy, chewing gum, sugared almonds, ice pops, liquor ice, sorbet, chocolate, chocolate wafer biscuits, chocolate caramel bars, chocolate bars, chocolates/bonbons/bon-bon, drinking chocolate/cocoa, white chocolate, milk chocolate, milk chocolate with nuts, dark chocolate, dark chocolate with nuts, cooking chocolate, chocolate hazelnut spread, chocolate truffles, nougat, marzipan, hard nougat, soft nougat, apple pudding/apple sauce, sweet quince jelly/quince jelly, caramel coated almonds.

Sauces and dressings
Garlic mayonnaise, ketchup, olive oil mayonnaise, sunflower oil mayonnaise, soya oil mayonnaise, mayonnaise, light mayonnaise, mustard, blue cheese sauce/Roquefort sauce, barbeque sauce, béarnaise sauce, béchamel sauce, Bolognese sauce, carbonara sauce, almond sauce, soya sauce, hollandaise sauce, mornay sauce, gravy, cocktail sauce, tartar sauce, green sauce, parsley sauce, industrial fried tomato sauce, sweet and sour sauce.

Pre-cooked dishes
Frozen fried floured squid, fried pastries baked meats (frozen), fried pastries, frozen baked cheese, frozen bread crumbed fish, fried spring rolls (frozen), frozen croquettes, frozen bread crumbed chicken, fried bread crumbed ham with cheese, frozen meat cannelloni with béchamel, frozen lasagna, frozen tomato and cheese pizza, canned squid in spicy tomato sauce, canned meat ravioli with tomato sauce, canned leek soup, canned

bean stew with Spanish sauce, canned gazpacho (cold Spanish tomato soup), hotdogs with mustard, cheese hamburgers with buns, hot ham and cheese sandwiches, croquettes, meats in pastry, cod fritters, meat and vegetable stock/soup, fish stock/soup, vegetable stock/soup, homemade cannelloni with béchamel, tomato soup, ham (chicken or meat croquettes), fish croquettes, tuna pastries, meat pastries, chicken soup with noodles, packed soup.

Salty snacks
Packaged snacks, corn snacks, salted crackers, salted cheese crackers, crackers, salted popcorn (with oil), popcorn (without oil), potato crisps/chips, low-fat potato crisps/chips, flavoured corn snacks, wotsits, Cheetos.

Non-alcoholic beverages
- *Instant coffee and cocoa*: cocoa/powdered drinking chocolate, instant decaffeinated coffee, instant coffee, eko/instant malt coffee substitute without water.
- *Soft drinks*: beverage/soft drinks/sodas, lemonade, sparkling lemon and lime flavoured soft drinks, sparkling cola soft drinks, sparkling diet cola soft drinks, sparkling lemon flavoured soft drinks, still lemon flavoured soft drinks, sparkling orange flavoured soft drinks, still orange flavoured soft drinks, tonic water, lemon flavoured slush puppy.
- *Packaged juices:* apricot juice/apricot nectar juice, exotic fruit juice/exotic fruit nectar juice, packaged mango nectar juice, packaged passion fruit nectar juice, packaged orange nectar juice, packaged pear nectar juice, packaged exotic fruit juice, packaged lime juice, packaged apple juice, packaged orange juice, packaged pear juice, packaged pineapple juice, packaged grapefruit juice, packaged tomato juice, packaged grape juice, packaged carrot juice.
- *Other non-alcoholic drinks*: isotonic drinks, bitter soda soft drinks without alcohol, alcohol free beer, powdered almond milk, tiger nut milk, grape juice, fruit juice, energy drinks.

Alcoholic drinks/Liquors
Bitter soda with alcohol, sangria, vermouth, sweet black vermouth, sweet wine/dessert wine, alcoholic liqueur, alcoholic fruit liqueur, clear fruit liqueur, dark fruit liqueur, sloe gin/sloe brandy, schnapps, dry anise, brandy/cognac, baileys/Irish cream, gin, rum, tequila, vodka, whisky/whiskey.

Miscellanea
Sweetener, jelly.
